# Strategies for Dodging the Obstacles in CAR T Cell Therapy

**DOI:** 10.3389/fonc.2021.627549

**Published:** 2021-04-01

**Authors:** Pooria Safarzadeh Kozani, Pouya Safarzadeh Kozani, Fatemeh Rahbarizadeh, Shahryar Khoshtinat Nikkhoi

**Affiliations:** ^1^ Department of Medical Biotechnology, Faculty of Medical Sciences, Tarbiat Modares University, Tehran, Iran; ^2^ Department of Medical Biotechnology, Faculty of Paramedicine, Guilan University of Medical Sciences, Rasht, Iran; ^3^ Student Research Committee, Medical Biotechnology Research Center, School of Nursing, Midwifery, and Paramedicine, Guilan University of Medical Sciences, Rasht, Iran; ^4^ Research and Development Center of Biotechnology, Tarbiat Modares University, Tehran, Iran; ^5^ Department of Pharmaceutics, Rutgers, The State University of New Jersey, Piscataway, NJ, United States

**Keywords:** chimeric antigen receptor, immunotherapy, tumor microenvironment, toxicities, adoptive cell therapy, solid tumors

## Abstract

Chimeric antigen receptor (CAR) T cell therapy has offered cancer patients a new alternative therapeutic choice in recent years. This novel type of therapy holds tremendous promise for the treatment of various hematologic malignancies including B-cell acute lymphoblastic leukemia (B-ALL) and lymphoma. However, CAR T cell therapy has experienced its ups and downs in terms of toxicities and efficacy shortcomings. Adverse events such as cytokine release syndrome (CRS), neurotoxicity, graft rejection, on-target off-tumor toxicities, and tumor relapse have tied the rescuing hands of CAR T cell therapies. Moreover, in the case of solid tumor treatment, CAR T cell therapies have not yielded encouraging results mainly due to challenges such as the formidable network of the tumor microenvironments (TME) that operates in a suppressive fashion resulting in CAR T cell dysfunction. In this review, we tend to shine a light on emerging strategies and solutions for addressing the mentioned barriers. These solutions might dramatically help shorten the gap between a successful clinical outcome and the hope for it.

## Introduction

Chimeric antigen receptor (CAR) T cells are genetically engineered T cells that possess the ability to specifically recognize and target tumor cells with significant discrimination from healthy tissues. Unlike the conventional cancer treatment methods such as surgery, radiotherapy, and chemotherapy, CAR T cells target tumor-specific antigens (TSAs) or tumor-associated antigens (TAAs) expressed on the surface of their target tumor cells with the delicate specificity granted to them by their antibody fragment-equipped targeting domain in a non-major histocompatibility complex (MHC) manner (1, 2).

In detail, CARs are made of an extracellular domain comprised of a targeting domain and a hinge, a transmembrane (TM) domain, and an intracellular domain composed of one or more co-stimulatory domains and an activation domain. The targeting domain of CARs are commonly composed of a single-chain variable fragment (scFv) derived from a monoclonal antibody (mAb) but other targeting domains such as single variable domains of heavy-chain antibodies (VHH, also known as Nanobodies^®^), ligands, and toxins have also been used, even though less commonly (2–4). Researchers have demonstrated the potential of VHH-based CAR T cells against solid tumors and in targeting their tumor microenvironments (TME) (3, 5). Moreover, in an interesting twist, Wang and colleagues used chlorotoxin as their CAR targeting domain because of its potential binding capacity to antigens associated with glioblastoma and they reported acceptable tumor elimination alongside undetectable off-target effects towards healthy cells, even *in vivo* (4). The authors also concluded that the reactivity of the chlorotoxin-equipped CAR T cells with their target cells is dependent on the expression of matrix metalloproteinase 2 (4).

So far, CD8, CD28, IgG1, and IgG4 have been used as hinges connecting CAR targeting domains to the TM domain. The TM domain of CARs is derived from molecules such as CD3ζ, CD8α, CD4, CD28, and the inducible T cell co-stimulator (ICOS) (6). Moreover, co-stimulatory domains are important components of CARs since they can contribute to different properties of CAR T cells (7–9). To this date, CD28, CD137 (4-1BB), CD134 (OX40), ICOS, CD27, MYD88-CD40, and KIR2DS2 have been used as co-stimulatory domains in various studies (10–12).

**Figure 1:**
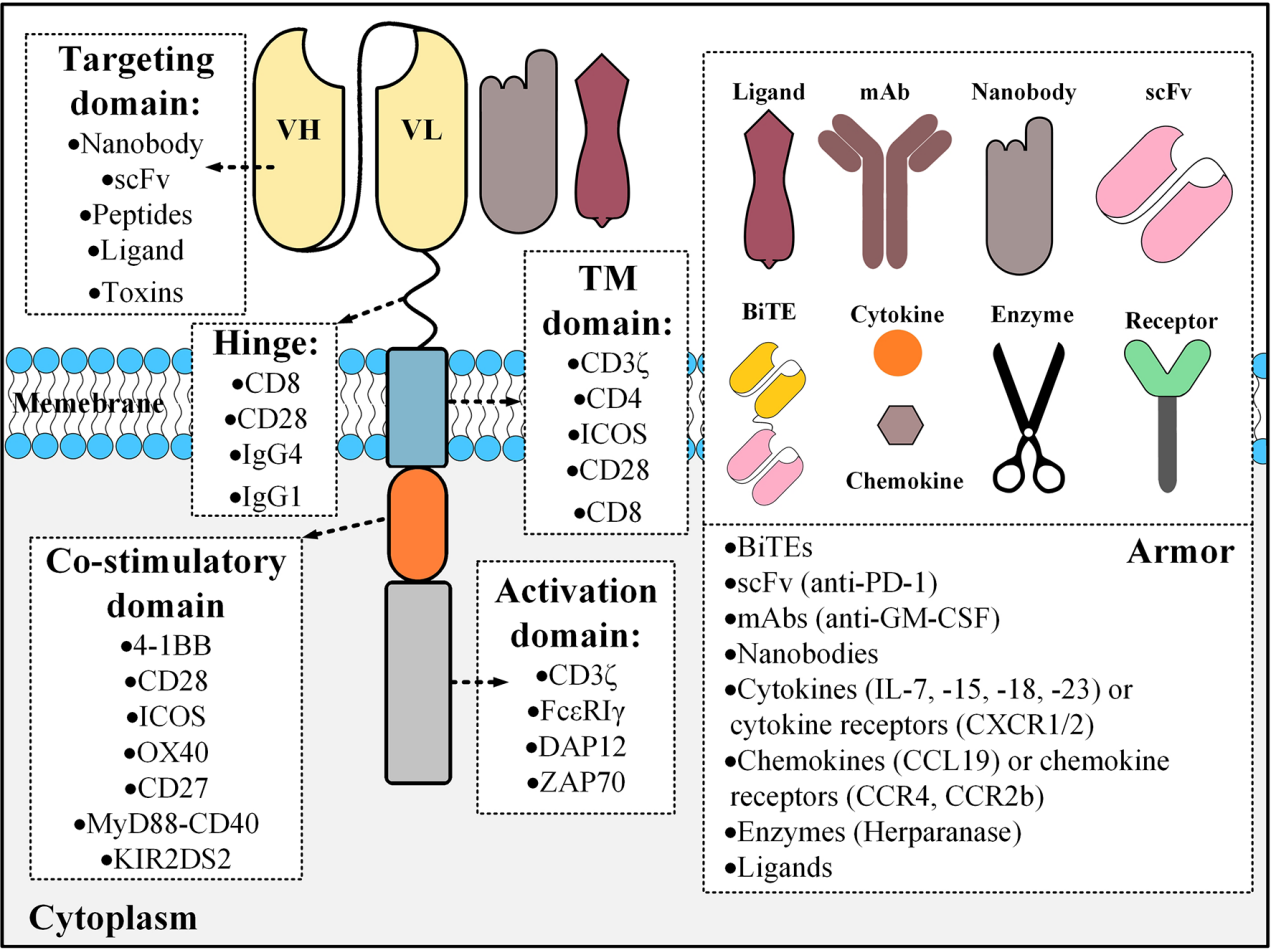
The building blocks of a CAR molecule and examples of different components that could be used for its construction. CAR constructs can also be engineered for the expression of an “*armor*” molecule that can operate in different aspects including the modifications of the tumor microenvironments, enhancing the homing of CAR T cells to the tumor site, or having immunomodulatory effects resulting in the augmentation of CAR T cell tumoricidal efficacy. Different types of armors are shown in the right panel with examples in parentheses. BiTE, bispecific T cell engager; TM, transmembrane; mAb, monoclonal antibody; scFv, single-chain variable fragment; ICOS, the inducible T cell co-stimulator; ZAP70, the ζ-chain of TCR-associated protein kinase 70 kDa.

The activation domain used in the structure of CARs has the critical role of T cell activation upon target antigen encountering and it is mostly derived from the CD3ζ part of the T cell receptor (TCR) CD3 complex (13). However, other activation domains that have been used include FcϵRIγ, the ζ-chain of TCR-associated protein kinase 70 kDa (ZAP70), and DAP12 (13–17). The early CAR T cells (termed “first-generation CARs”) did not have any co-stimulatory domain and did not show promising antitumor efficacy mainly due to the lack of adequate persistence and activation (18). For addressing these caveats, researchers added co-stimulatory domains to the intracellular domain of CARs generating “second-generation” and “third-generation” CARs which have one and two co-stimulatory domains, respectively (13). These CAR T cells showed enhanced persistence, activation, and effector function in clinical trials in comparison with first-generation CARs (19, 20). Researchers have even stepped further by adding an inducer domain of a specific cytokine such as interleukin (IL)-2 to the intracellular domain of second-generation CARs only to generate T cells redirected for universal cytokine-mediated killing (TRUCKs) or armored CARs (21). These CAR T cells can deliver transgenic products or payloads to the targeted tumor tissues leading to the enhancement of antitumor activity and efficacy of CAR T cell therapy (21). Additionally, according to Kershaw et al., the activation and proliferation of T cells might be more favorable if accompanied by a third cytokine engagement signal besides the other two primary activation and co-stimulation signals (22). In this regard, Kagoya1 et al. redecorated second-generation CARs, originally harboring the CD3ζ activation domain and CD28 co-stimulation domain, by adding a truncated cytoplasmic domain from the IL-2 receptor β (IL-2Rβ) that harbors a STAT3-binding site (23). The *in vitro* investigations of these researchers revealed that the activation of the JAK kinase, STAT3, and STAT5 pathways are dependent on the engagement of the CAR with its target antigen (23). Moreover, these novel CAR T cells were capable of establishing more outstanding persistence and tumoricidal activity in preclinical models of hematologic and solid tumors compared with their conventional counterparts (23). **Figure 1** represents a detailed description of the various components of CARs.

CAR T cell therapy has induced remarkable clinical outcomes, especially in the treatment of hematologic malignancies, which has led to the US Food and Drug Administration (FDA) approval of four CD19-targeting CAR T cell products, named *Kymriah*™ (*Tisagenlecleucel*), *Yescarta™* (*Axicabtagene ciloleucel*), *Tecartus™* (*Brexucabtagene autoleucel*), and *Breyanzi™* (*Lisocabtagene maraleucel*) (24–27). So far, patients with conventional treatment-resistant relapsed/refractory (R/R) B-cell Acute Lymphoblastic Leukemia (B-ALL), Diffuse Large B Cell Lymphoma (DLBCL), and Mantle cell lymphoma (MCL) can only profit from the therapeutic benefits of the mentioned products (24–27).

However, from the early days of CAR T cell therapy, it has been accompanied by various types of side effects, which can range from manageable mild to irreversible life-threatening toxicities. Such toxicities created various safety concerns that have limited the broader application of CAR T cells in various oncological indications. Therefore, understanding their nature and developing strategies for their mitigation are subjects of paramount importance. Neurologic toxicity and Cytokine Release Syndrome (CRS) (characterized by the overproduction of immune-regulatory cytokines and factors) alongside immune responses mediated by the recipient’s immune system against the infused CAR T cells (recognized as foreign invaders) have been rather frequent in the related clinical settings (28, 29). In the case of CD19-based CAR T cell therapies, the long-term persistence of CAR T cells that fail to discriminate between malignant and healthy B cells leads to the elimination of the latter, long after treatment completion (28, 30). This phenomenon is known as B cell aplasia and it renders the respective patients susceptible to infections caused by numerous bacteria (28, 30). Additionally, due to the poor availability of TSAs, a high proportion of CAR T therapies target TAAs that are also expressed by normal cells at physiological levels (29). Since such CAR T cells fail to discriminate between normal and malignant cells, there have been cases of serious adverse events against healthy tissues (which are known as on-target off-tumor toxicities) (29). Furthermore, disease relapse has also been observed both in the cases of blood-based malignancies and solid tumors (31). This occurrence is a result of target antigen loss or extreme antigen downregulation which is undertaken by tumor cells as a potential mechanism for immune evasion (31). Ultimately, the harsh immunosuppressive features of the TME can also impinge on CAR T cell functionality, in the context of solid tumors (32). In this review, we tend to present a detailed description of the mentioned caveats and then discuss intelligent strategies aimed at removing them. Furthermore, we brief how such elaborate strategies might enable a safer and more efficient CAR T cell therapy.

## Fighting CRS and Neurotoxicity Mediators

CRS is the most common side effect of CAR T cell therapy that is usually observed several days following the adoptive transfer (28, 33). CRS is commonly characterized by elevated levels of IL-1, IL-2, IL-6, IL-8, IL-10, interferon-γ (INF-γ), granulocyte-macrophage colony-stimulating factor (GM-CSF), and tumor necrosis factor α (TNF-α) in a patient’s serum (28, 33). Patients experiencing CRS usually manifest hypotension, fever, pulmonary edema, and hypoxia (28). However, depending on the grade and severity of CRS, other symptoms could also be observed in the affected patients including tachycardia, myalgias, diarrhea, nausea, acute kidney injury, anemia, arrhythmias, and hyperbilirubinemia (28). Such manifestations could be resultant from CRS-related damages to various vital organs of the patients which require meticulous medical care (28). The starting point of this so-called storm is the activation of CAR T cells following their engagement with their target antigen (28, 33). This activation leads to the production and secretion of inflammatory cytokines by CAR T cells (28, 33). In response to these cytokines, other innate immune cells, such as macrophages, begin to release inflammatory cytokines such as IL-1 and IL-6, thus creating a loop of inflammation (28, 33). To take the situation under control, the mentioned self-intensifying loop needs to be disrupted.

In contrast with CRS, the rate of neurotoxicity in CAR T therapies has been rather inconsistent in different reports (28, 34). Patients with neurologic toxicity often experience seizures, hallucinations, delirium, brain edema, headache, or tremor (28, 34). To this date, the exact mechanism that gives rise to CAR T cell-induced neurotoxicity remains a mystery (28). However, investigators have reported the presence of CD19-redirected CAR T cells in the patients’ cerebrospinal fluid alongside elevated levels of pro-inflammatory cytokines (28, 34). Moreover, a high concentration of such cytokines might activate the endothelium of the brain vessels and the blood-brain barrier (BBB) resulting in their permeabilization and consequent cerebral edema (35). Based on a recent report by Parker and colleagues, in the case of CD19-based CAR T cell therapies, the observed neurotoxicity could be attributed to the targeting of CD19-expressing brain mural cells by CAR T cells (36). Since mural cells provide vital support for the BBB, their elimination might facilitate CAR T cell infiltration into the brain (hence a high possibility of cerebral edema emergence) (36). In this section, we briefly discuss strategies that could be beneficial in the management of CAR T cell-mediated CRS and neurologic toxicity.

### GM-CSF Blockade

GM-CSF is a macrophage- and monocyte-activating cytokine known to be an important factor in mediating CRS (28, 29). GM-CSF can be neutralized using mAbs, such as lenzilumab, which can result in a significant reduction of myeloid and T cell infiltration in the central nervous system (CNS) (37). This reduction has been helpful in the mitigation of neuroinflammation (NI) and the prevention of CAR T cell-mediated CRS in preclinical models (37). Additionally, not only this method does not interfere with CAR T cell functionality, but it also elevates their tumoricidal efficacy by reducing the risk of CAR T cell-mediated CRS and NI (37). In detail, GM-CSF neutralization inhibits the secretion of CRS-causing cytokines such as IL-6 and reduces the production of other CRS-mediating pro-inflammatory factors including IL-8 and monocyte chemoattractant protein 1 (MCP-1), which act as immune cell trafficking mediators (38, 39). In addition to antibody-mediated neutralization, genetic engineering methods can also be used for the manipulation of CAR T cells that are less likely to mediate CRS and neurotoxicity (37, 40). Recently, studies have shown that knocking out the GM-CSF gene in CAR T cells using transcription activator-like effector nucleases (TALEN) or CRISPR/Cas9 can significantly reduce the production and secretion of GM-CSF, which can consequently abrogate the macrophage-dependent secretion of CRS-associated biomarkers such as MCP-1, IL-6, and IL-8 (37, 40). Also, this approach has reduced the levels of key CRS-mediators and enhanced the antitumor activity of CAR T cells in preclinical models (37). Above all, CAR T cells can also be genetically engineered to secrete GM-CSF neutralizing antibodies which can further mitigate the risk of CRS and neurotoxicity.

### IL-1 and IL-6 Blockade

Studies have shown that monocyte- and macrophage-released IL-1 and IL-6 are associated with CAR T cell-mediated CRS and immune effector cell-associated neurotoxicity syndrome (ICANS) (41, 42). Preclinical data indicate that monocytes are a more responsible source of IL-1 and IL-6 during CRS occurrence (42). It has been demonstrated that CRS can be prevented by methods such as monocyte ablation or IL-6 receptor blockade using tocilizumab (42). However, it has been reported that tocilizumab does not prevent delayed lethal neurotoxicity in preclinical mouse models (42). In this case, Anakinra, which is an immunosuppressive drug and an IL-1 receptor antagonist, has shown promising results after administration into preclinical mouse CRS models by protecting them from both lethal neurotoxicity and CRS (42). Of note, anakinra can be as effective as tocilizumab in rescuing preclinical mouse models from lethal CRS (42). Furthermore, other studies have reported that IL-6 receptor blockade may not be completely sufficient in controlling severe CRS and it might be necessary to use high-dose corticosteroids for this aim (43, 44). Other studies have engineered CAR T cells to secrete IL-1 receptor antagonists which have induced promising effects in preventing or reducing CRS and neurotoxicity in preclinical mouse models (41). In conclusion, IL-1 and IL-6 are both key players in the development and progression of post-CAR T cell infusion CRS and neurotoxicity (42). Targeting strategies against IL-1 can be an applicable approach for the prevention and mitigation of both CAR T cell-induced CRS and neurologic toxicities.

### Catecholamine Blockade

Recently, it has been found that high levels of circulating catecholamines can mediate various types of immune-dysregulation, including CRS, through a self-augmenting loop in macrophages (45). Catecholamines have effective roles in the release of cytokines induced by T cell-activating therapeutic agents (45). It has been found that inhibition of catecholamine synthesis can result in a significant reduction in the level of cytokine release both *in vitro* and *in vivo* (45). Also, it has been demonstrated that atrial natriuretic peptide (ANP) can reduce the levels of circulating catecholamines without interfering with the tumoricidal activity of CAR T cells (45, 46). Furthermore, myeloid-secreted catecholamines are also known as critical mediators of CRS (45). Researchers have indicated that myeloid-specific ablation of tyrosine hydroxylase (an essential enzyme involved in the synthesis of catecholamines) using metyrosine can protect mouse models of lymphoma with stimulated macrophages from lethal complications of CRS after CD19-based CAR T cell therapy (45). Such studies indicate that catecholamines are key modulators of cytokine release, and not only blocking their synthesis pathway does not lead to side effects or CAR T cell functionality impairment but it also might reduce the incidence of CRS development and progression (45, 46). Such tactics also suggest that the modification of cellular pathways involved in CRS progression might reduce the risk of this life-threatening toxicity (45).

## Fighting Immune Rejection

From the emerging days of CAR T cell therapy, the pros and cons of the cell sources, from which CAR T cells are generated, have been under investigation. Using autologous T cells (derived from the patients themselves) for producing CAR T cells is not always feasible because of the patients’ disease burden or the particular treatment course they are under. On the other hand, allogeneic T cells (obtained from healthy donors) are not completely limitation-free as they might be rejected by the recipients’ immune system (28, 29). This unfavorable event is mostly mediated by the recipient’s T cells and natural killer (NK) cells, as these cells recognize the allogeneic CAR T cells as invading foreign cells that should be eradicated from the host’s body (47–51).

### Alloimmune Defense Receptor (ADR)

One of the most recent strategies for addressing the issue of allogeneic CAR T cell rejection exploits the 4-1BB cell surface receptor present on the recipients’ T and NK cells (51). The expression of this receptor is upregulated in activated T cells and NK cells (51). This strategy uses an engineered receptor named Alloimmune Defense Receptor (ADR), which is made of a 4-1BB-recognizing domain derived from 4-1BB ligand (4-1BBL), an intracellular CD3ζ domain, a spacer, and a transmembrane domain (51). The ADR is designed to be expressed on the surface of CAR T cells (51). In detail, the ADR recognizes the upregulated 4-1BB molecule on the surface of activated alloreactive T cells and NK cells which leads to the activation of the ADR-expressing CAR T cells and results in the elimination of the recipients’ alloreactive immune cells (51). Moreover, ADR expression does not impinge on the effector function of CAR T cells, therefore, this approach can give allogeneic CAR T cells a new weapon that they can use against immune cells trying to interfere with their fight against tumors (51).

**Figure 2:**
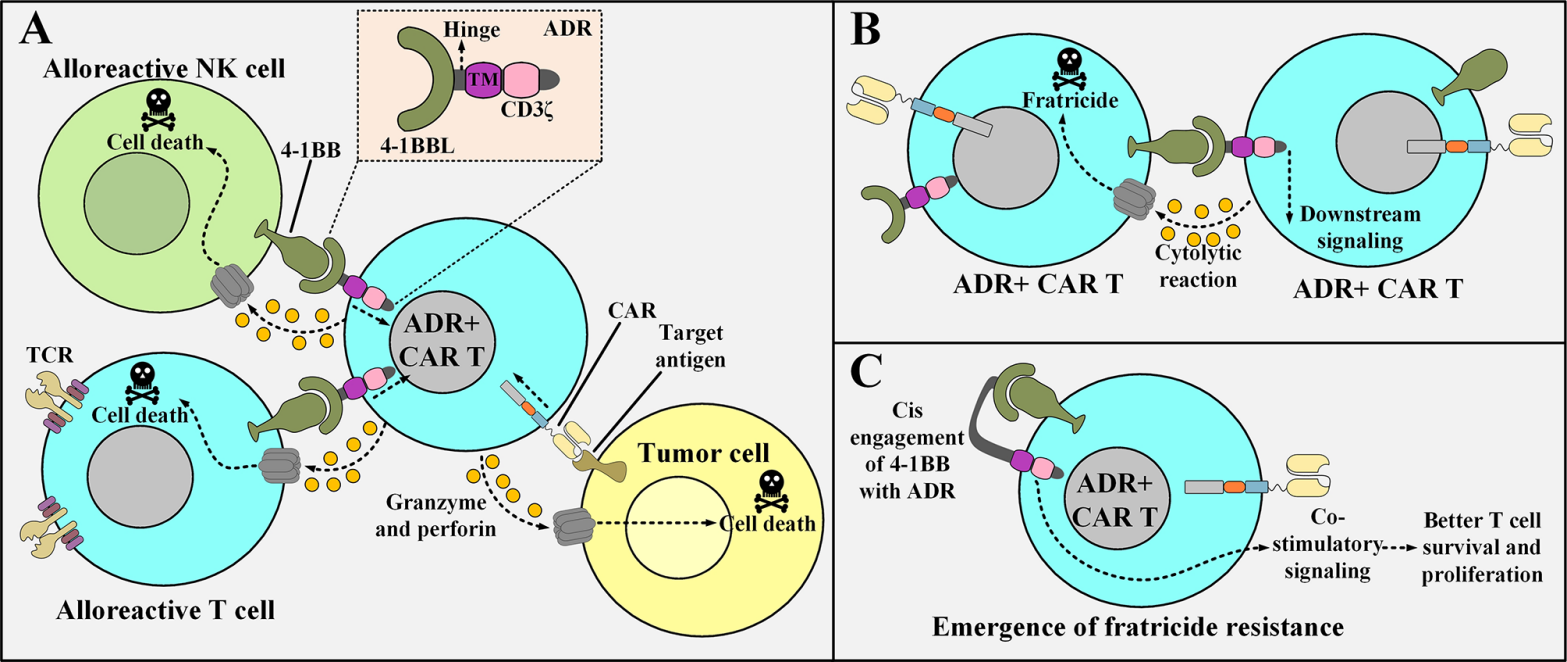
The underlying of mechanism of action of ADR-expressing CAR T cells, the issue of their fratricide, and the possible mechanism of action for the emergence of fratricide-resistant ADR-positive CAR T cells. **(A)** ADR-expressing CAR T cells enforce cytolytic reactions only against alloreactive T cells and NK cells (that are activated with their 4-1BB upregulated) and manage to spare resting T cells and NK cell (that are non-alloreactive). On the other hand, they can also enforce tumoricidal responses against their target tumor cells. **(B)** Following their activation, ADR-positive CAR T cells upregulate 4-1BB. This makes them susceptible to fratricide (self-cytotoxicity). **(C)** A proposed underlying mechanism for the emergence of ADR-expressing CAR T cells that are resistant to fratricide. The surface expressed ADR reacts with the 4-1BB molecule that is upregulated after the activation of the ADR-positive CAR T cells. This phenomenon renders 4-1BB hidden from being recognized by other ADR-expressing CAR T cells (hence the ADR-expressing CAR T cell represented in the figure becomes fratricide-resistant). TM, transmembrane domain; 4-1BBL, a fragment derived from the 4-1BB ligand; ADR, alloimmune defense receptor; NK, natural killer; CAR, chimeric antigen receptor; TCR, T cell receptor.

However, since ADR-expressing CAR T cells upregulate 4-1BB expression (following activation), they might become subjects of fratricide (self-cytotoxicity) (52). Mo et al. also reported limited fratricide both in preclinical models and *in vitro* (however, transient) (52). An interesting mechanism could be attributed to this limited fratricide based on similar situations already observed in other studies (52). Ruella and colleagues reported that accidental transduction of leukemic B cells (during the manufacturing process of CD19-redirected CAR T cells) might give rise to the resistance of such leukemic cells to CD19-based CAR T cell therapy (52). The underlying mechanism for this resistance is that the transduced leukemic B cells start to express the CD19-specific CAR molecule which eventually manages to engage with their CD19 antigen (52). This self-reactivity leads to the masking of the CD19 epitope recognized by the CD19-redirected CAR T cells, therefore, the CAR-expressing leukemic B cells evade the antitumor reactions of the mentioned CAR T therapy (52). This scenario might somehow be possible in the case of activated ADR-positive CAR T cells as it helps them become fratricide-resistant (51). In detail, following activation, ADR-positive CAR T cells start to upregulate 4-1BB on their surface which consequently manages to engage with their ADR (51). This self-engagement masks the 4-1BB molecule, therefore, it can no longer be recognized by other ADR-positive CAR T cells (the emergence of fratricide-resistant ADR-positive CAR T cells) (51). Furthermore, this self-engagement might also provide the mentioned fratricide-resistant CAR T cells with amplified proliferation and persistence signals (51, 53, 54). **Figure 2** represents a detailed description of ADR-expressing CAR T cells.

### CD47 Expression

CD47 is a transmembrane protein responsible for mediating a “*do not eat me*” signal in numerous types of malignant cells (55). The signal regulatory protein-α (SIRPα) is recognized as the receptor for CD47 on various immune cells including macrophages (55). Once tumor cells that express the CD47 antigen on their surface encounter macrophages, CD47 binds to SIRPα which leads to the transmission of the “*do not eat me*” signal and consequent abrogation of phagocytosis by macrophages (55). Therefore, using this mechanism, malignant cells can easily evade immune system-mediated eradication (55). This mechanism can be applied when using allogeneic CAR T cells to avert macrophage-assisted CAR T cell rejection and subsequent clearance. In this regard, CAR T cells can be engineered to express CD47 on their surface as a means of evading phagocytosis by macrophages.

### TCR and HLA Knock Outs

Investigators have also exploited genetic engineering methods for diminishing the level of alloreactivity when using allogeneic CAR T cells. The TRAC gene is one of the foremost targets in this regard whose knock-out using diverse genetic manipulation tactics, such as TALEN, zinc-finger nuclease (ZFN), and CRISPR-Cas9, has been established to be effective in the ablation of both TCR α and β chains and alleviating alloreactivity (56–60). Furthermore, other researchers have highlighted the use of allogeneic CAR T cells with TCR and CD52 knock-outs and they have demonstrated that these cells act as satisfactory universal CAR T cell candidates since they do not cause alloreactivity and are able to mediate molecular remission in patients with R/R B-ALL (57). It is worth mentioning that the knock-out of CD52 renders these CAR T cells resistant to the depletion impacts of the anti-CD52 antibody alemtuzumab (57). Additionally, CRISPR-Cas9-mediated CAR transgene knock-in in the TRAC gene locus has also been investigated by other researchers as they have suggested that this method can also be as efficient as the other mentioned methods for disrupting the endogenous TCR expression in allogeneic CAR T cells (61). Also, CRISPR-Cas9 and ZFN both have been utilized for the ablation of HLA expression to diminish the level of alloreactivity when using allogeneic CAR T cells as well (62, 63).

## Strategies for Overcoming On-Target Off-Tumor Toxicity

TAAs targeted by CAR T cells are usually expressed by healthy tissues as well, even though at lower rates. However, despite this limited target antigen expression, CAR T cells still manage to recognize these normal cells and initiate cytolytic reactions against them. This phenomenon results in the elimination of those healthy cells (known as “on-target off-tumor” toxicities), thus causing life-threatening side effects such as multi-organ failure for the respective patients. B cell aplasia, as characterized by the elimination and absence of B cells, is a renowned off-tumor incident after CD19- or CD22-based CAR T cell therapy mediating hypogammaglobulinemia in the recipients that subjects them to various types of infectious diseases (30, 64). In detail, CD19 and CD22 are both expressed on normal B cells as well as the malignant ones (64). Therefore, CAR T cells targeting either of these antigens happen to eradicate normal B cells as well (64). In this regard, B cell aplasia is considered as a parameter for the assessment of CD19 and CD22 CAR T cell therapy efficacy and persistence as well as their success rate (64). It can also act as a marker for the possibility of disease relapse (64). To tackle these limitations, scientists have engineered smart CAR constructs with tumor-selectivity mechanisms capable of precise discrimination between malignant and healthy cells. In this section, we will briefly discuss some of these strategies alongside highlighting their advantages and disadvantages.

### Masked CARs

**Figure 3:**
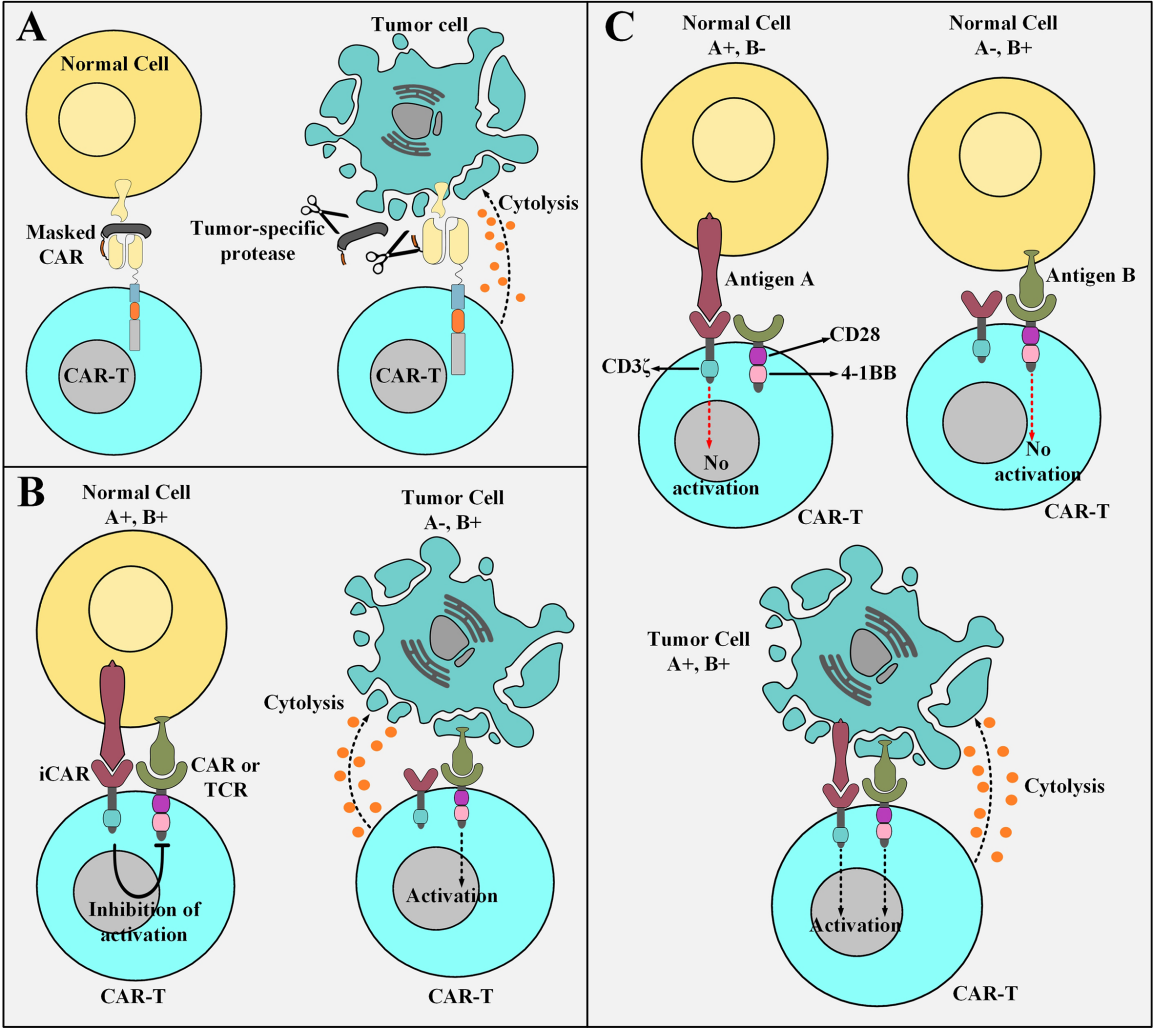
Elaborate strategies for tackling the on-target off-tumor toxicity of CAR T cells. **(A)** Masked CAR T cells and their mechanism of action. Masked CAR T cells are unable to cytotoxically attack healthy cells because their targeting domains are veiled. In the tumor microenvironment, the masking peptide is dissociated due to the presence of tumor-specific proteases. This phenomenon results in the recognition of the tumor cells by masked CAR T cells leading to the cytolysis of the tumor cells. **(B)** iCAR T cells and their mechanism of action. iCAR T cells are cytotoxically inert while encountering healthy cells simultaneously expressing their inhibitory and activatory antigens. Upon encountering tumor cells that only express the activatory antigen and are deficient in the expression of the inhibitory antigen, iCAR T cells are activated to carry out their tumoricidal effects. **(C)** The logic-gated CAR platform and its mechanism of action. Logic-gated CAR T cells require the presence of two antigens for their activation and triggering of cytotoxic effects. This strategy increases their safety index while encountering healthy tissues expressing one of the antigens. CAR-T, chimeric antigen receptor T cell; iCAR, inhibitory CAR; TCR, T cell receptor.

TME exhibit an upregulated expression profile for multiple classes of tumor-associated proteases such as plasmin (65, 66), matrix metalloproteases (67), cathepsins (68–70), and legumain (71, 72) which could be exploited to engineer smart CAR platforms. A conditionally active CAR construct whose antigen-recognition domain is composed of a probody constitutes the novel strategy of “masked CARs” which increase the applicability of CAR T cells in the treatment of cancers that lack definitive TAAs (73). In detail, a probody is an antibody with its antigen recognition site covered by a masking peptide recombinantly linked to it by a protease-sensitive linker that is susceptible to proteolytic cleavage only by TME proteases (73, 74). Conceptually, the protease-sensitive linker is cleaved in the presence of tumor-associated proteases leading to the subsequent disengagement of the masking peptide and unveiling of the antigen-binding site of the targeting domain (73). This occurrence opens the gate for the downstream tumoricidal responses of the effector cells (**Figure 3A**) (73). Probodies, as compared to conventional mAbs, have shown a tremendously increased safety index due to their prolonged pharmacokinetic half-life which enables them to reach higher exposure rates while dosed at the same level as that of conventional mAbs (74). This expanded safety zone might be translatable in the field of masked CAR T cell therapy in a way that higher infusion dosages can impose more effective therapeutic impacts without crossing the red line of safety (73). Furthermore, the use of universal linkers sensitive to several proteases secreted by different types of TME makes the masked CAR platform a reliable candidate for targeting multiple tumor types in a concurrent manner (73). Despite the quiescence of masked CAR T cells in the circulation until their trafficking into the TME, there are still some off-tumor toxicities delivered to healthy tissues that secret the proteases to which the linker peptide is sensitive (73). However, such occurrences do not yet defame the generosity and expanded safety profile of this CAR platform (73).

### Inhibitory CAR (iCAR)

Another strategy to minimize the deleterious damages of “on-target off-tumor toxicities” or bystander healthy tissue damages is the use of an antigen-specific iCAR. This platform provides a self-regulating dynamic safety switch to circumvent the consequences of unwanted T cell responses and divert them away from the undesired tissues (75). The general concept is to have a surface antigen recognition domain fused to the signaling domains of endogenous immunoinhibitory receptors such as CTLA-4 or PD-1 to reversibly restrict T cell cytokine secretion, cytotoxicity, and proliferation despite concurrent engagement of an activating receptor (which can be a CAR or simply just an engineered TCR) (75). The iCAR platform allows T cells to discriminate between healthy and cancerous cells in an antigen selective manner (**Figure 3B**) (75). The transgenic expression of the iCAR construct does not impinge on the basic functionality of the T cells in the absence of its specific inhibitory antigen (75). Moreover, other T cell-restricted inhibitory receptors such as BTLA, 2B4, and LAG-3 or their combination in a single second-generation iCAR or as an iCAR with multiple combined cytoplasmic domains can also be used to regulate the cytotoxic functionality of CAR T cells (75–77).

Despite being experimentally evident that iCAR T cells can still sustain their tumoricidal functionality even after exposure to inhibitory antigens, the possibility of a proportion of iCAR T cells becoming anergized over repeated exposure to an inhibitory antigen is not completely ruled out (75, 78). Moreover, since this elaborate regulatory approach is antigen-specific, it requires tissue-specific target antigens that are expressed by healthy tissues but are absent from or down-regulated by tumor cells (75). Human leukocyte antigen (HLA) can be a suitable antigen of such characteristics since it is expressed in all cell types but substantially downregulated by tumor cells to confer them the ability to escape T cell-mediated immune system responses (79).

### Logic-Gated CAR T Cells

A trans-signaling CAR strategy in which T cell activation signal and co-stimulatory signal are physically dissociated from each other in two antigen-specificity different CARs has been developed to equip CAR T cells with a “*double or nothing*” strategy (80–84). These CAR T cells (known as logic-gated CAR T cells) can only attack tumor cells that simultaneously express both of the antigens recognized by the antigen recognition domains of the two different CARs (hence they manage to spare healthy cell expressing only one of the antigens) (80–84). Conceptually, T cells are genetically modified to express two CARs; one CAR that only harbors the CD3ζ signaling domain and recognizes an antigen of interest with low affinity and a chimeric co-stimulatory receptor (CCR) that recognizes a different antigen of interest with high affinity (80–84). Moreover, CCR engagement with antigen provides the co-stimulatory signaling cascades necessary for T cell activation and potent cytotoxicity (80–84). Genetically modified T cells expressing these two constructs are not potently activated while they encounter normal cells, which only express one of the two antigens, due to insufficient activation signals (**Figure 3C**) (80–84). However, several issues question the practicality of this proposed strategy. Limitations such as identification of two tumor antigens which are only expressed by a given type of cancer with nonoverlapping expression in normal tissues (81). Additionally, another limitation relates to the difficulty of designing a CAR with a narrow optimum affinity range or a CAR that is practically applicable to almost a broad spectrum of patients (80). *In vitro* findings have demonstrated weak cytokine secretion by trans-signaling CAR T cells against cells expressing only one TAA and pronounced cytokine secretion upon encountering tumor cells co-expressing both antigens (81). These findings suggest that the dual-specificity trans-signaling CAR platform might potentiate the therapeutic efficacy of CAR T cells against target cancer cells while diminishing their cross-reactivity with normal tissues (81).

### γδ T Cells Harboring Co-Stimulation-Only CARs

**Figure 4:**
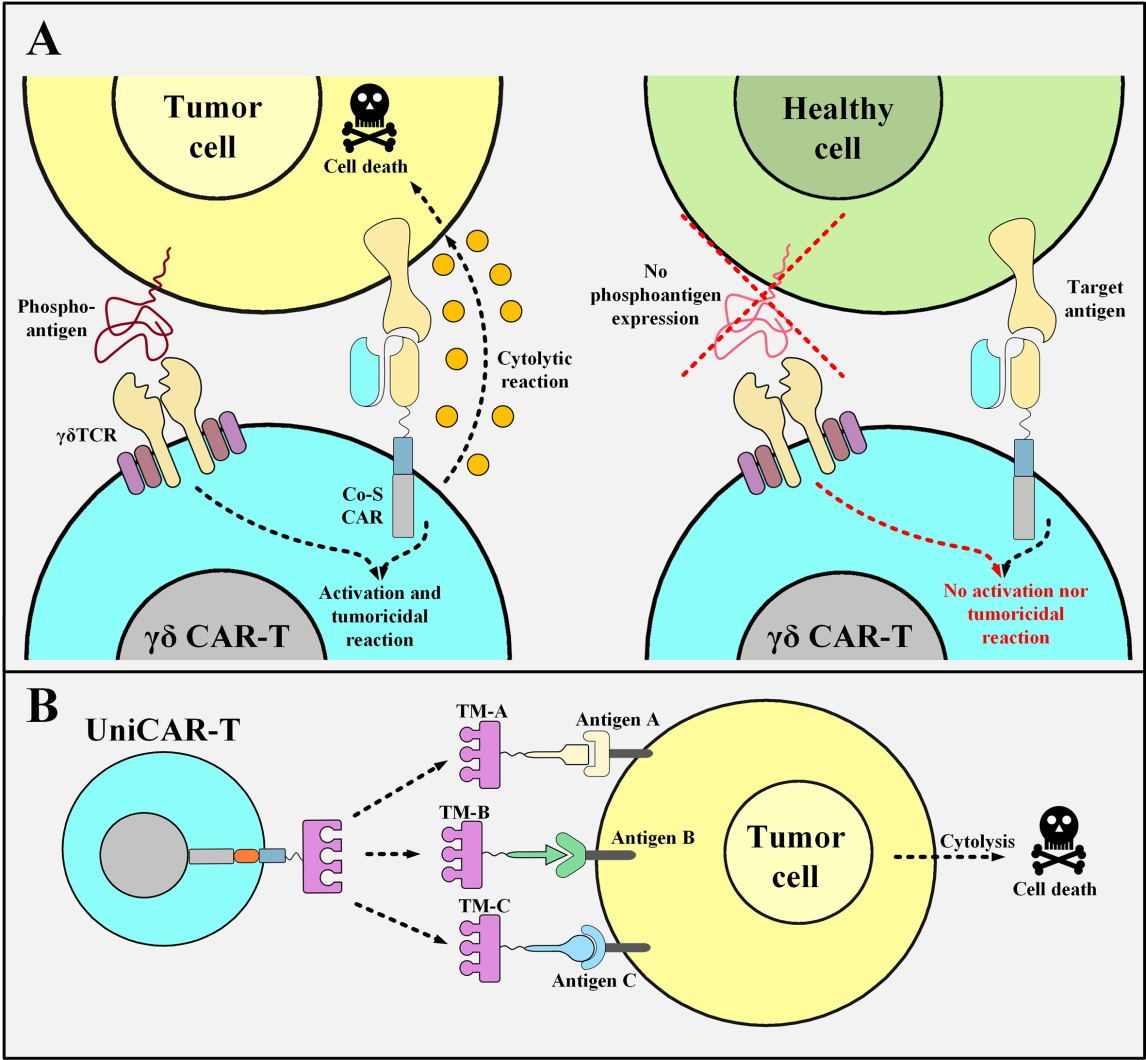
Co-stimulation-only γδ CAR T Cells and UniCAR T cells and their mechanism of action. **(A)** The mechanism of action of γδ CAR T cells with “co-stimulation-only” CARs and “Vγ9Vδ2 TCRs”. The tumoricidal activity of γδ CAR T cells will be in action when the CD3ζ activation and co-stimulation signals are provided. The activation signal is mediated through the Vγ9Vδ2 TCR of these CAR T cells only when they encounter the phosphoantigens expressed by the metabolically dysregulated tumor cells. Furthermore, the co-stimulation signal is provided once the co-stimulation-only CAR molecules recognize their specific TAA. Since healthy cells do not express such phosphoantigens, γδ CAR T cells will not be able to mediate cytotoxic reactions against them. **(B)** UniCAR T cells. UniCAR T cells can be activated and redirected towards different tumor cells based on the presence of different TMs. The clearance of the TM from the circulation simply results in the quiescence of the UniCAR T cells. Co-S CAR, co-stimulation-only CAR; TM, target module; UniCAR-T, universal chimeric antigen receptor T cell.

Recently, researchers have used γδ T cells, which are a subset of T cells that harbor TCRs with γδ subunits, instead of the more common αβ subunits (85, 86). γδ T cells are about 1-10% of circulating T cells but they act as important components of the immune system (87). Vγ9Vδ2 T cells, which are a subset of γδ T cells, have an intrinsic tumor-distinguishing ability since they can recognize the phosphoantigens that are non-peptidic tumor antigens and are typical features of metabolism-dysregulated tumor cells (88). Researchers have investigated a novel tactic using Vγ9Vδ2 T cells as the backbone for generating unique “co-stimulatory domain-only CARs” (89). Dissimilar from the conventional αβ T cells (used as the primary source for the production of CAR T cells), γδ T cells recognize their target antigens with no dependence on MHC class I or II (90). The Vγ9Vδ2 TCR is the most prevalent γδ TCR expressed by γδ T cells (90). These TCRs recognize phosphoantigens such as isopentenyl pyrophosphate (IPP) over-produced in cancer cells and not in healthy ones (90). γδ T cells differentiate between cancerous and normal cells by identifying these antigens as a “danger alarm” (90). Studies have demonstrated that GD2-targeting co-stimulation-only CAR T cells generated from T cells with Vγ9Vδ2 TCRs are functional and show robust cytolytic responses only against GD2-positive neuroblastoma cells, but not against GD2-positive normal cells, *in vitro* (89). This fact highlights the role of the endogenous Vγ9Vδ2 TCR since the CD3ζ signal is only provided by the tumor cells that interact with the endogenous Vγ9Vδ2 TCR (**Figure 4A**) (89).

Moreover, other studies have also presented similar promising results demonstrating that CAR T cells generated using Vδ2 T cells (termed γδ CAR T cells) can migrate towards tumor cells and perform antigen cross-presentation (91). These findings propose that γδ CAR T cells can enter the tumor site and eliminate tumor cells alongside uptaking the target antigens which lead to stimulatory antigen presentation to tumor-infiltrating lymphocytes (TILs) with αβ TCRs (91). It has been proposed that tumors such as melanoma can be the right battlefield for these fighting cells since they harbor high tumor antigen frequency and large numbers of tumor-reactive lymphocytes and TILs (91). This fact may be considered as an advantage of γδ CAR T cells over conventional CAR T cells which could be quite worthwhile in the treatment of solid tumors (91). Moreover, various studies have also indicated that graft-versus-host disease (GVHD) is mediated by alloreactive T cells harboring the αβ TCR, while T cells with the γδ TCR do not mediate alloreactivity, therefore they are not capable of GVHD induction (92). Besides, γδT cells also can orchestrate various compelling antileukemic and anti-infectious effects (92). However, the functionality of γδ CAR T cells in comparison with conventional CAR T cells, their in-detail characterization, and their large-scale manufacturing protocols are yet to be explored. In a nutshell, it can still be concluded that γδT CAR T cells might show promise for prospective clinical evaluations in solid tumors since they present exclusive useful properties over conventional CAR T cells.

### Universal CARs (UniCARs)

Another elaborate strategy to diminish the risk of off-tumor side effects entails the use of a modular CAR platform known as UniCAR. This strategy makes it possible to reversibly turn off the CAR system as fast as possible in case of unwanted CAR T cell-mediated side effects (93). UniCARs are designed as two different components that form an immune complex together to be guided towards the desired target cells. Conceptually, UniCARs consist of the UniCAR effector T cells and engineered recombinant target modules which direct them to the surface of the appropriate target cells (**Figure 4B**) (93). The specificity of the target module dictates the UniCAR T cells exactly which target cells they should attack and their rapid elimination from the circulation sufficiently vouches for their safety index as it switches UniCAR T cells “On” and “Off” (93). Since the antibody domain of the UniCAR T cells is directed against a unique epitope on the target module, they can establish an immune complex in their presence (93). This would guide UniCAR T cells towards their target cells (93). On the other hand, in the absence of a target module, UniCAR T cells automatically turn off which makes their controlling much more feasible than that of conventional CAR T cells (93–95). To minimize the considerable risks of CRS during a UniCAR T cell therapy, the administration of a rapidly eliminated target module should start at low doses and then be adjusted and increased based on the emergence of unexpected side effects (93). Once the desired target cells are eliminated or any life-threatening adverse events happen, the termination of targeting module administration will simply cause the UniCAR T cells to be turned off (93). Additionally, the potential benefits of the UniCAR platform in the cases of disease relapse are discussed in an upcoming section.

## Strategies for Overcoming the Post-Infusion Control Limitation

So far, various attempts have been made for controlling the activity of CAR T cells after their infusion into patients. This topic deserves special attention since it can contribute to the control and prevention of the previously mentioned CAR T cell-mediated toxicities which can sometimes be life-threatening. In this section, we briefly summarize strategies aimed at controlling the expression of CARs on the surface of the engineered T cells as well as some of the most potent strategies developed for overall control over CAR T cells after their administration.

### The Lymphocyte-Specific Protein Tyrosine Kinase (LCK) Inhibition

It has been demonstrated that the tyrosine kinase inhibitor Dasatinib, an FDA-approved treatment for Philadelphia chromosome-positive chronic myeloid leukemia (CML) and ALL, inhibits LCK and thereby prevents the phosphorylation of CD3ζ and ZAP70 (96). Mestermann and colleagues have exploited dasatinib to increase the safety index of CAR T cells (96). The mentioned mechanism can mediate the disruption of the downstream signaling cascade in CARs harboring either CD28-CD3ζ or 4-1BB-CD3ζ activation modules (96). Moreover, dasatinib can induce a quickly occurring (3 hours) hybernation in CD8- and CD4-positive CAR T cells which can continue for several days without imposing any negative effects on T cell viability (96). Moreover, different dosing schemes of dasatinib can be used for partial or complete inhibition of CAR T cell activity (96). It has been shown that the administration of dasatinib shortly after CAR T cell infusion in preclinical CRS mouse models protects them from CRS that could otherwise be lethal in models not receiving dasatinib (96). The main advantage of this method is that upon the discontinuation of dasatinib administration, its inhibitory effect rapidly and completely reverses, therefore, the previously affected CAR T cells can continue their normal signaling pathway and antitumor activity (96). The favorable pharmacodynamics of dasatinib is another advantage of this approach allowing for multiple-time utilization of this drug for sequential CAR T cell activity turning “off” and “on”. Conclusively, dasatinib administration in CAR T cell-receiving preclinical models and *in vitro* assays pauses cytolytic activity, cytokine production, and expansion of CAR T cells and it can be applied as a pharmacologic on/off switch for CAR T cells (96). Aside from these, this approach might suffer from several limitations. One is that the inhibitory impact of dasatinib over CAR T cells that have already been activated is less pronounced (96). Furthermore, since dasatinib exerts its inhibitory effect through TCR signaling, the endogenous T cells will also be affected in terms of their effector function (96, 97). Another limitation of this approach affects patients with aggressive tumors whose malignant cells tend to proliferate at a high speed (96). In such patients, over the course of toxicities, while dasatinib is administered to control CAR T cell functionality, the CAR T cell-mediated tumoricidal reactions are halted temporarily which leaves more room for tumor progression (96).

### STOP CAR

**Figure 5:**
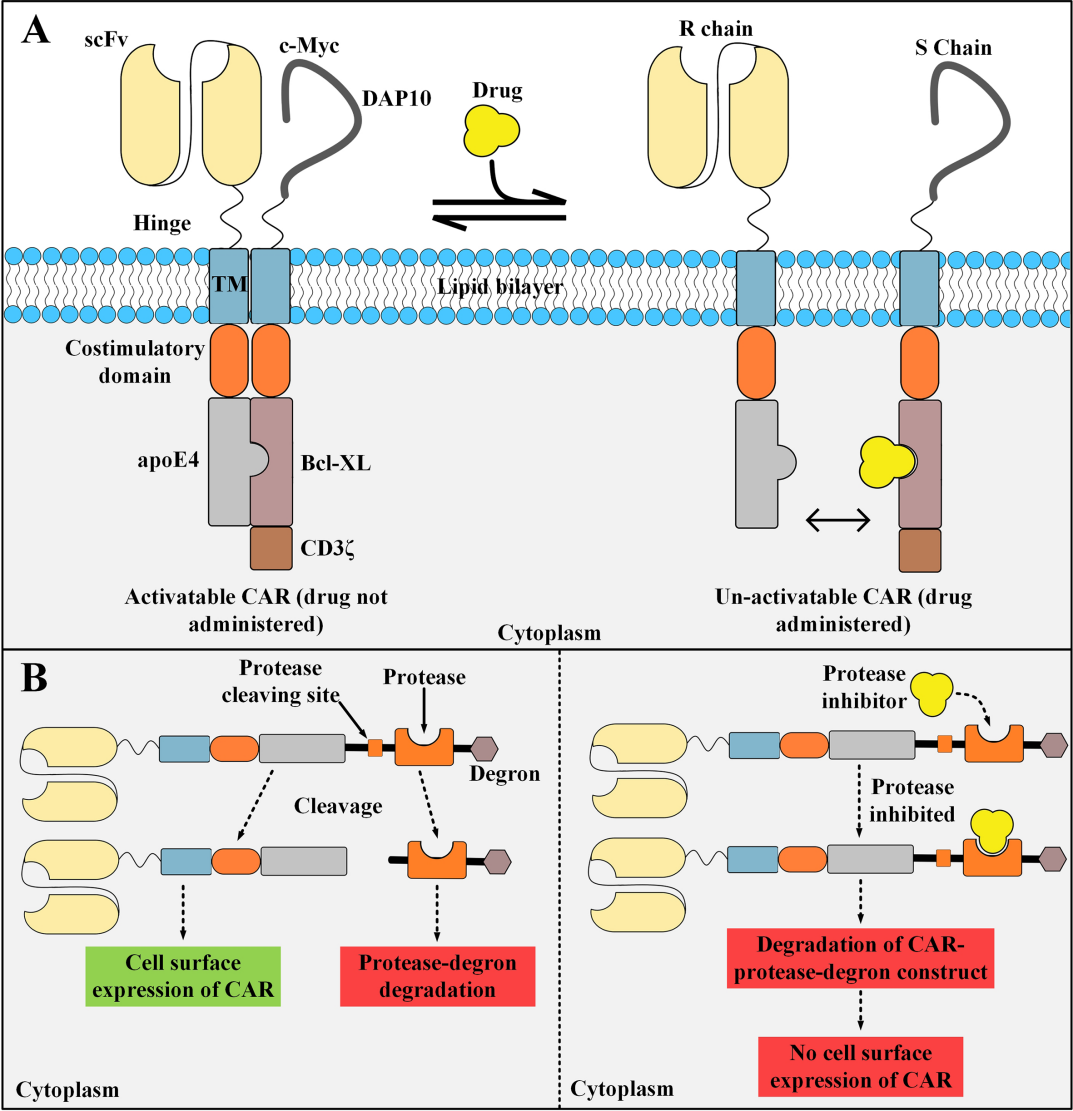
STOP CAR and SWIFF-CARs structures and action mechanisms. **(A)** The STOP-CAR platform. The S chain is made of a c-myc and DAP10 (which are used for enhancing the stability and expression of the S chain), a hinge, a transmembrane domain, a co-stimulatory domain, Bcl-XL, and CD3ζ. On the other hand, the R chain is made of an scFv, a hinge, a transmembrane domain, a co-stimulatory domain, and human apolipoprotein E4. When the disruptive drug is not administered, the R and S chain can bind to each other and the CAR can be activated upon target antigen engagement. After the disruptive drug administration, it binds to its binding site on the Bcl-XL domain located on the S chain, therefore, it renders the R and S chain unable to pair and the CAR un-activatable. **(B)** A simplified illustration of the SWIFF-CAR platform. In the absence of the protease inhibitor (left panel), the protease retains its proteolytic activity and binds to its cleaving site leading to the dissociation of the CAR molecule from the rest of the construct and its translocation to the cell surface resulting in normal CAR-mediated antitumor activity. In the presence of the protease inhibitor (right panel), the inhibitor binds to its binding site located on the protease and mediates its inhibition, therefore, the protease cannot bind to its cleaving site which results in the degradation of the CAR molecule and the rest of the construct in the T cell proteolytic pathways (right panel). apoE4, human apolipoprotein E4; scFv, single-chain variable fragment; CAR, chimeric antigen receptor. R chain, recognition chain; S chain, signaling chain.

STOP CAR is a recently developed CAR platform made of a recognition (R) chain, responsible for antigen binding, and a signaling (S) chain, responsible for T cell activation (98). The endodomains of these two distinct chains have a computationally designed protein pair that helps them dimerize into a functional heterodimer without the need for a dimerizing agent (98). This heterodimer is a chemically disruptable heterodimer (CDH) and it can be exclusively disrupted and dissociated into two monomers by the administration of small molecules such as A1331852 and A1155463 (which are Bcl-XL inhibitors) (98). The availability of disruptive small molecules that have valid clinical applications, prolonged half-life, and significant tolerance in humans are the principles for the design of such CDHs (98). The basic aim of the STOP CAR platform is to utilize globular domains from modular proteins that do not disturb the synapse-proximal T cell signaling (98). In detail, the CDH is made of human apolipoprotein E4 (apoE4), which is located on the R chain, and Bcl-XL, which is located on the S chain (98). apoE4 and Bcl-XL are human-originated proteins with very few numbers of amino acid substitutions (98). Therefore, they might not lead to transgene immune rejection in the recipients receiving these CAR T cells (99, 100). This strategy enables us to decrease the activity of the infused CAR T cells temporarily instead of shutting it down permanently (which happens in the case of suicide switches, as discussed in the upcoming subsections) (98). When there are no disruptive small molecules administered, apoE4 and Bcl-XL are paired together allowing CAR T cell activation upon the recognition of the antigen of interest (98). On the other hand, in the presence of the disruptive small molecules, apoE4 and Bcl-XL are dissociated from each other and maintain their monomeric form which does not allow CAR T cell activation upon antigen recognition (**Figure 5A**) (98). STOP CAR T cells have been tested against two different antigens (PSMA and CD19), and it has been found that their efficacy is equal to their respective conventional second-generation CAR T cells (98). Taken together, STOP CARs can be utilized for controlling the post-infusion effector function of CAR T cells in a very safe, cost-effective, reversible, and efficient manner (98). However, when such protein engineering techniques are applied for the development of a CAR molecule, there is a slight possibility for the emergence of immunogenic epitopes (98). In this regard, elaborate computational approaches can be used for the depletion of such T cell epitopes (98, 101).

### SWIFF-CARs

The activity of CAR T cells can be controlled through mechanisms that can regulate the expression of CAR molecules on the surface of T cells (as an on- and off-switch). Recently, Juillerat et al. have generated a CAR T cell activity-controlling platform termed switch-off CARs (SWIFF-CARs) which entails using the protease-based small molecule-assisted shutoff (SMASh) (102). In this platform, the SWIFF-CAR construct is made of the CAR molecule followed by a protease cleaving site, a protease (the HCV NS3 protease), and a degradation moiety named “degron” (102). In the absence of the cell-permeable protease inhibitor Asunaprevir, the protease cleaves its target site leading to dissociation of the CAR from the protease and degron (102). This change will result in the translocation of the CAR molecule to the cell surface allowing its normal activity and signaling cascade (a state called “ON”) (102). On the other hand, in the presence of asunaprevir, it binds to its binding site on the protease and inhibits its cleaving activity (102). Therefore, the CAR molecule will not dissociate from the protease cleaving site, protease, and degron which will lead to the proteolytic degradation of the CAR molecule (a state called “OFF”) (**Figure 5B**) (102). This study shows that it might be feasible to directly incorporate an off-switch into the CAR construct which enables reversible control of the CAR surface expression (102). However, this tactic is just considered as an *in vitro*-tested prototype of a seemingly applicable idea for now. In-depth *in vivo* preclinical investigations may better highlight the applicability of CAR T cells equipped with this switch in terms of *in vivo* expansion and tumoricidal activity as well as the ability to discriminate the healthy cells from the malignant ones.

### Suicide Strategies

Selective and permanent ablation of CAR T cells in emergencies including the occurrence of GVHD or on-target off-tumor toxicities has been the subject of numerous investigations over the past years. The need for safety switches capable of irreversible elimination of CAR T cells during the mentioned adverse events and the implementation of such strategies have been recognized as an efficient way for addressing these challenges. One of these safety switches is based on suicide gene technologies which function through different mechanisms such as metabolic pathways, agent dimerization as well as targeting *via* therapeutic mAbs. These switches are discussed in detail throughout the upcoming section. It is worth mentioning that biological quiescence alongside favorable bioavailability and biodistribution profiles are all among the desired characteristics of an ideal suicide switch activation agent (103).

#### Metabolic switches

**Figure 6:**
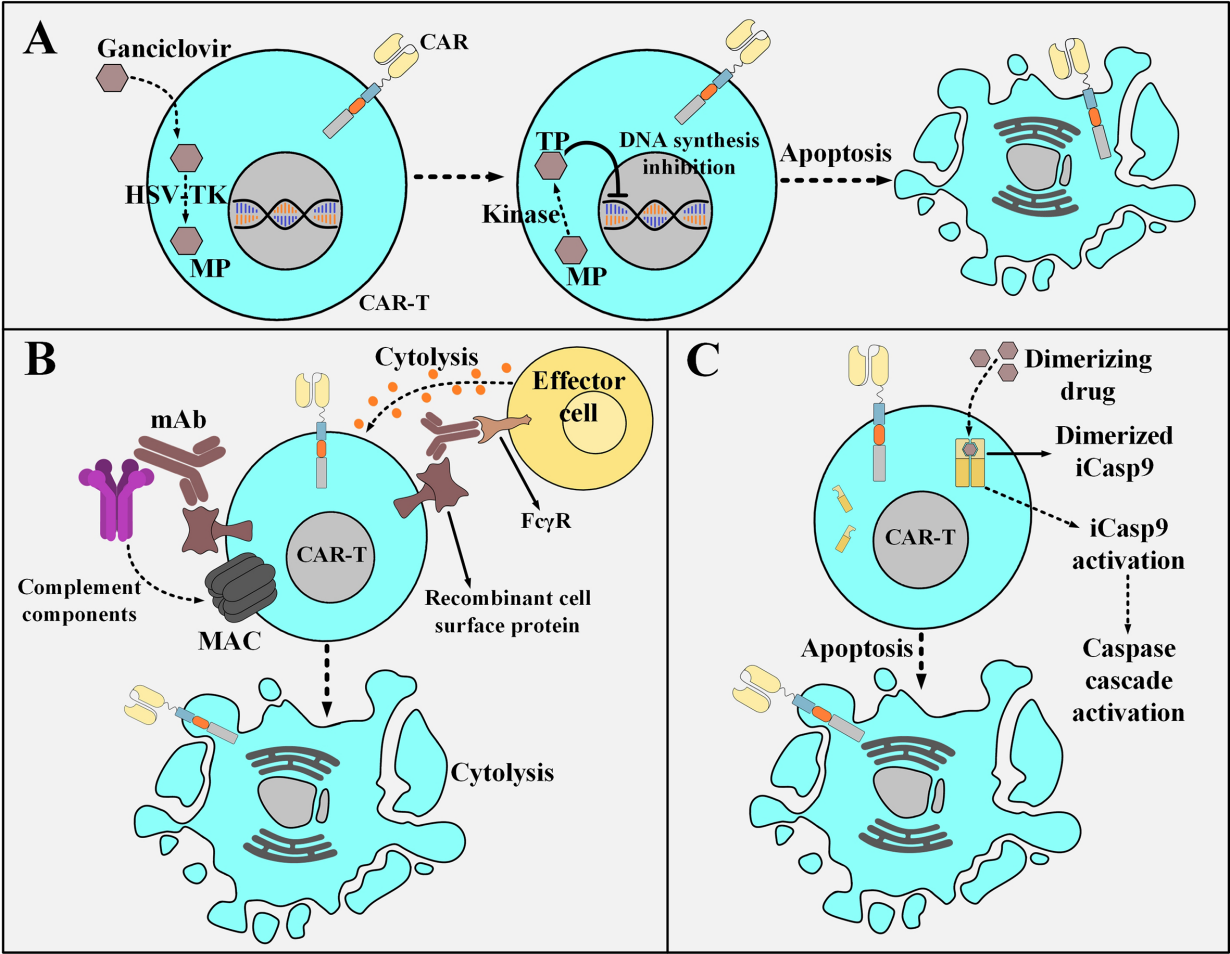
Various strategies for the post-infusion controlling of CAR T cells. **(A)** The HSV-TK safety switch and its mechanism of action. Upon the administration of ganciclovir, it is catalyzed to MP by HSV-TK. MP will eventually be phosphorylated to TP. Subsequently, TP disrupts DNA synthesis leading to the selective ablation of CAR T cells. **(B)** An example a mAb-based safety switch and its mechanism of action. Co-expression of a desired recombinant cell surface protein enables selective elimination of CAR T cells expressing that molecule through the administration of pharmaceutical-grade mAbs which are specific for it. This elimination is mediated by the engagement of immune effector cells or complement fixation. **(C)** The iCasp9 suicide switch and its mechanism of action. The administration of the dimerizing drug results in the dimerization of the iCasp molecule. The dimerized iCasp is now activated and can trigger downstream apoptotic cascades that result in the death of the CAR T cells harboring this switch. HSV-TK, Herpes simplex virus thymidine kinase; MP, ganciclovir-monophosphate; TP, ganciclovir-trisphosphate; MAC, membrane attack complex; mAb, monoclonal antibody; FcγR, Fcγ receptor; iCasp9, inducible caspase 9.

Suicide switches can be based on converting a non-toxic compound into a toxic one which eventually acts to kill the suicide switch-harboring cell. Herpes simplex virus thymidine kinase (HSV-TK), unlike mammalian cell thymidine kinase, shows an incredibly high affinity to ganciclovir (GCV), which is a nucleoside analog (104, 105). GCV is phosphorylated by HSV-TK to GCV-monophosphate (MP) and which is eventually converted to GCV-trisphosphate (TP). DNA polymerase incorporates GCV-TP into the leading strand of DNA which results in GCV-induced chain termination (106, 107). The HSV-TK/GCV suicide switch is also capable of triggering death-inducing signaling cascades through the formation of Fas-associated death domain protein (FADD) and the activation of caspases through ligand-independent CD95 aggregation (108). Despite the gradual effectiveness and potential immunogenicity risks of the HSV-TK switch (due to its viral origin), its benefit-to-risk ratio might still be clinically favorable (**Figure 6A**) (104, 105). Another example of this type of suicide-inducing switch involves cytosine deaminase (CD), which is a pyrimidine salvage enzyme (109). Mechanistically, 5-fluorouracil (5-FU) is the product of the deamination of the antifungal medication 5-fluorocytosine (5-FC) by CD, and therefore it plays the role of the highly cytotoxic compound capable of cell death induction (109). In this regard, equipping CAR T cells with the genes encoding enzymes such as HSV-TK or CD enables irreversible elimination of the infused CAR T cells in times of encountering adverse complications. Furthermore, type 1 HSV-TK gene has also been known as a positron emission tomography (PET) reporter gene that can be leveraged for providing insights into CAR T cell trafficking into tumor sites (110).

#### mAb-Based Switches

Another suicide switch strategy for the selective *in vivo* ablation of CAR T cells is their genetic engineering to coordinately express a CAR and a recombinant cell surface protein (111–113). This recombinant protein should retain a conformationally intact binding epitope recognized by a given pharmaceutical-grade mAb such as cetuximab (EGFR-specific mAb) or rituximab (CD20-specific mAb) (111–113). This approach renders the mentioned CAR T cells susceptible to antibody-dependent cell-mediated cytotoxicity (ADCC) or complement-dependent cytotoxicity (CDC) upon exposure to that reagent without altering their cytotoxic functionality (**Figure 6B**) (111–113). The suicide system based on the use of cetuximab can be considered a more compatible option for application in patients with hematologic malignancies (including ALL) since it would not ablate B cells and it is also a more considerate option for those leukemia patients who are seeking adoptive T cell therapy but are already under rituximab treatment. Moreover, there has not been any truncated form of the tetraspan transmembrane protein of CD20 that can conformationally retain its capacity for rituximab binding (112, 114–116). It is also encouraging to mention that there have been no signs of immunogenicity mounted against the EGFRt molecule (a truncated form of the epidermal growth factor receptor) of the EGFRt-positive CAR T cells in preclinical mouse models (113). These findings support the hypothesis that the administration of cetuximab might result in the recovery of the B cell compartment in patients who have undergone CD19-based CAR T cell therapy and experienced prolonged persistence of CD19-redirected CAR T cells, B cell aplasia, and complete tumor regression (113). However, there are still some concerns about the clinical applicability of such mAb-based switches since the administration of mAbs into patients might result in serious damages to healthy tissues that express the native form of the recombinant protein (103). Even though such concerns might limit the broader development of safety switches based on this platform, their clinical evaluation completed in the upcoming years will elucidate these matters.

#### iCasp

Another example of an inducible safety switch is based on the recombinant fusion of a modified FKBP12 (human FK506-binding protein) to the human caspase 9 or the membrane-anchored intracellular domain of Fas (117, 118). This approach enables at-will dimerization in the presence of a biologically inert dimerizing agent (such as AP1903) (117, 118). Conceptually, the modified FKBP12 binds to the synthetic dimerizing drug with high affinity allowing for the dimerization and subsequent activation of the inducible caspase 9 (iCasp9) or the Fas-based suicide switch (117, 118). This results in a caspase cascade that leads to the apoptosis of the cells expressing these constructs (**Figure 6C**) (117, 118). Furthermore, studies have reported the elimination of >90% of the iCasp9*-*equipped T cells within 30 minutes following a single-dose administration of the dimerizing drug in patients with GVHD (118). Moreover, this rapid elimination correlated with the resolution of GVHD without recurrence (118). Furthermore, the iCasp9 switch has been known to possess several advantages over other safety switches such as its low immunogenicity profile (due to the human origin of the iCasp9 suicide gene) and the utilization of a biologically inert small molecule for its activation (instead of antiviral agents such as ganciclovir) (118). These advantages make this safety switch a more suitable option for application in the field of cellular therapy. Moreover, the rapid cell death mediation of this switch through the engagement of the endogenous apoptotic pathway in the cell (occurring minutes after the administration of the dimerizing drug) is much faster than other safety switches that require interference with DNA synthesis for cell death induction (119–124)

## Strategies for Overcoming Tumor Relapse

No single antigen might be considered a universal one because of the antigenic heterogeneity profile within a single tumor and among different patients. Antigen loss, antigen downregulation, or the emergence of alternatively spliced antigens (that no longer can be targeted by CAR T cells due to the loss of the recognized epitope) are all among elaborate antigen-dependent strategies performed by tumor cells to escape immune recognition (125–127). Such phenomena consequently limit the tumoricidal efficacy of targeted immunotherapy resulting in poor clinical responses (125–127). Simultaneous multispecific targeting is one of the proposed strategies aimed at offsetting tumor antigen escape variants which might provide enhanced durability of immunotherapy-mediated remission. CAR T cells whose CAR constructs are equipped with bispecific targeting domains in a tandem manner (Tandem CAR or TanCAR) or T cells co-expressing two different chimeric receptors specific for two distinct TAAs might have superiorities compared with conventional CAR T cells. These genetically manipulated T cells are endowed with the ability to cytotoxically target tumor cells expressing either antigen or both antigens simultaneously. Such CAR T cells have exhibited accentuated antitumor activity *in vitro* and in animal models of human tumors such as glioblastoma and B cell malignancies (128–134). According to a recent report from a clinical trial (NCT03185494) evaluating the tumoricidal efficacy of bispecific CD19/CD22-redirected CAR T cells in adult R/R B-ALL patients, all of the 6 patients (100%) experienced MRD-negative CR without the onset of neurotoxicity (135).

Furthermore, in cases of disease relapse, UniCAR T cells can also be beneficial since they can initiate cytotoxic reactions against the evading tumor cells upon the introduction of targeting modules that target a new tumor antigen (rather than the ones alternatively spliced or with expression loss or downregulation) (93). This capability demonstrates why this CAR platform is universally applicable towards different target antigens of interest without the need for redesigning a new CAR construct.

## Strategies for Overcoming the Immunosuppressive TME

### The Hypoxic TME Nature

**Figure 7:**
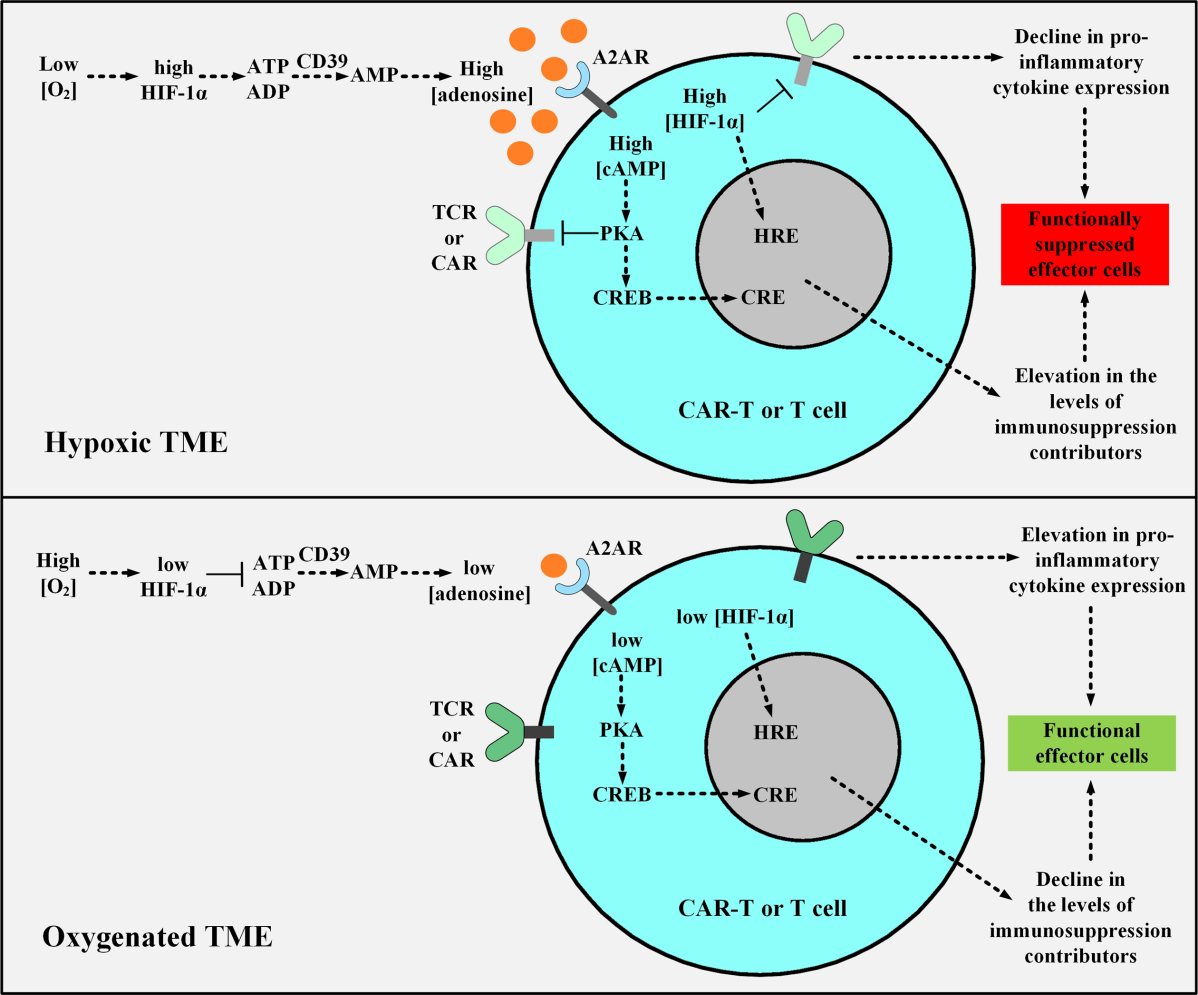
The effects of hypoxic or oxygenated TME on the fate, functionality, and antitumor activity of T cell or CAR T cells. The upper panel shows the hypoxic TME. In this condition, the low level of O_2_ leads to the stabilization of HIF-1α which elevates the expression level of CD39 and CD73 leading to the excessive production of adenosine. Adenosine binds to A2AR causing an elevation in the level of cAMP, and cAMP activates PKA. Further on, PKA inhibits TCR and CAR signaling and upregulates the expression of immunosuppresion contributors via CRE. Moreover, HIF-1α elevates the expression level of immunosuppressive genes through HRE. The activated CRE and HRE signaling cascades together reduce the level of INF-γ, IL-12, and IL-2 expression and upregulate TGF-β, IL-10, PD-1, CTLA-4, COX-2, and Treg expression. In the lower panel, supplemental oxygenation or using oxygenation agents elevates the level of O_2_, therefore, the downstream signaling pathway cannot proceed as it would in the case of a hypoxic microenvironment. TME, tumor microenvironment; HIF-1α, Hypoxia-inducible factor-1α; A2AR, A2A adenosine receptor; PKA, protein kinase A; CRE, cAMP Response Element; CREB, cAMP Response Element-Binding Protein; HRE, Hypoxia Response Element; TCR, T cell receptor; CAR, chimeric antigen receptor.

The major differences in the nature of normal and cancerous tissues can be exploited for developing smart TME-responsive or -dodging therapeutic approaches. The poor level of nutrition availability, low extracellular pH (acidosis), and low oxygenation level (hypoxia) are among various TME-specific characteristics (136, 137). The hypoxic microenvironment is characterized by oxygenation levels often below 1-2% (136, 137). Moreover, the immunosuppressive hypoxia-A2-adenosinergic axis is a very interesting characteristic of many treatment-resistant tumors (138). Discovering the key roles of the upstream factors in this pathway has led to the development of unique counterstrategies for inhibiting the hypoxia/hypoxia-inducible factor-1α (HIF-1α) axis (139, 140). Preclinical investigations targeting the A2A adenosine receptor (A2AR) and the adenosine-generating ectoenzyme CD73 have led to significant therapeutic efficacy (139–142). In detail, the hypoxic environment of the TME stabilizes HIF-1α leading to an elevation in the expression level of adenosine-generating ectoenzymes including CD39 and CD73 (143). The increments in the level of CD39 and CD73 elevate the level of adenosine (143). Further on, adenosine binds to A2AR on T cells which leads to the elevation in the level of cAMP (143). This elevation activates the cAMP-dependent protein kinase A (PKA), through the binding of cAMP to PKA (143). The downstream signaling cascade resulting from PKA activation in T cells leads to the blockage of TCR signaling and the expression of immunosuppression-responsible genes through the cAMP Response Element (CRE) (143). HIF-1α additionally controls the expression of factors responsible for tumorigenicity and immunosuppression through the Hypoxia Response Element (HRE) in a straightforward manner (143). Therefore, the CRE and HRE downstream signaling cascades decrease the level of INF-γ, IL-12, and IL-2 secretion and elevate the level of the TGF-β signaling pathway and IL-10, PD-1, CTLA-4, COX-2, and T-regulatory (Treg) expression (143). Conclusively, the hypoxic nature of the TME mediates an elevated level of anergy and exhaustion and a reduced level of cytokine production and secretion in T cells and CAR T cells (143). Researchers have shown that supplemental oxygenation and utilizing oxygenation agents can reverse hypoxia in the TME (143–145). They have suggested that this method can avert the stabilization of HIF-1α and impair the hypoxia-adenosinergic immunosuppressive axis (143–145). They have demonstrated that this method can reprogram the nature of the TME from “*immunosuppressive*” to “*immunopermissive*” (143–145). Moreover, they have underlined the clinical application of systemic oxygenation and oxygenation agents in conjunction with the A2AR blockade to further tackle the TME immunosuppressive nature (143). This strategy disrupts the upstream and downstream (hypoxia-HIF-1α and adenosine-A2AR, respectively) cascades of the immunosuppressive hypoxia-adenosinergic signaling axis and can maximize the therapeutic benefits of A2AR antagonists alongside elevating the susceptibility of tumors to cancer treatments (**Figure 7**) (143). Furthermore, other researchers have exploited the hypoxia of TME and have designed smart self-decision-making CAR T cells (146). They have fused an oxygen-sensitive subdomain of HIF-1α to a CAR scaffold and generated CAR T cells that are responsive to a hypoxic environment (146). This strategy has been developed to restrict the expression of CAR to only those CAR T cells residing in the hypoxic TME (rather than the ones in the non-hypoxic environment of non-malignant tissues) (146). Therefore, these CAR T cells can reduce the off-tumor effects of conventional CAR T cells (146).

### Metabolic Reprogramming of CAR T Cells

The effector function and differentiation state of T cells are highly impacted by their cellular metabolism condition (147). Moreover, the components of CARs expressed in transduced T cells have impacts on their nutritional intake and metabolic state (147). The metabolism-functionality relationship found in T cells can be used as a tool for defining their fate, activity, and effector function (147). For instance, studies have shown that the presence of 4-1BB co-stimulatory domain in the construct of CARs persuades the T cells to develop central memory phenotype and have an enhanced oxidative breakdown of fatty acids alongside improving their expansion capacity and persistence (147). On the other hand, the CD28 co-stimulatory domain improves glycolysis and makes CAR T cells develop effector memory phenotype (147). Moreover, supplementation such as supplementing with L-arginine can balance the elevated arginine metabolism in activated T cells alongside improving tumoricidal functionality and inducing central memory phenotype development (148).

Having a detailed gene expression profile of the genes mostly involved in cellular metabolism can help us achieve the goal of metabolic reprogramming of T cells by modifying the expression level of metabolic genes. This topic has been at the center of T cell reprogramming investigations since it has recently shown encouraging results. In this regard, it has been found that leukemic cells inhibit the Akt/mTORC1 signaling of T cells triggering their impaired functionality (149). Leukemic cells also mediate the downregulation of the glucose transporter Glut1 leading to a decreased level of glucose uptake in T cells (149). Overexpressing the Akt pathway or the Glut1 transporter is the proposed strategy to tackle this caveat caused by leukemic cells (149). This strategy can somewhat bring back the functionality of the T cells to the level before the negative impacts were imposed on them by the tumor cells (149). Moreover, PPAR-gamma co-activator 1a, also known as PGC1a, is a transcription factor co-activator affecting various cellular metabolic pathways. This metabolic regulator is downregulated in T cells infiltrating tumor sites (150). Researchers have found that the overexpression of PGC1a in T cells rebuilds their effector functionality as well as their metabolic and mitochondrial activity (150). Additionally, genetically suppressing Acetyl-CoA acetyltransferase (ACAT1), which is a cholesterol esterification enzyme converting excess cholesterol to cholesterol esters, in T cells leads to an elevation in the cholesterol concentration of the T cell plasma membrane (151). As a consequence, this phenomenon will effectively improve T cell signaling, thus mediating a better effector function and antitumor activity (151).

Furthermore, studies have used the characteristics and behavior of tumor cells and tissues to reprogram the metabolism of T cells (152, 153). In this regard, it has been discovered that necrotic tumor cells release potassium (K^+^) in the TME which leads to the excessive accumulation of this ion (152). This phenomenon elevates the intracellular concentration of K^+^ in the tumor-infiltrating T cells more than the normal level leading to a limitation in their nutrient uptake (152). Moreover, this accumulation in T cells downregulates their Protein Kinase B (Akt)/mammalian target of rapamycin (mTOR) signaling and interferes with T cell activation signaling (152). Researchers have shown that overexpressing K^+^ channels can act to reduce the elevated intracellular K^+^ levels, promote Akt/mTOR activity, and bring back the diminished effector function of T cells (152). Moreover, researchers have found a solution for the K^+^ accumulation-mediated limited nutrient uptake of T cells in the TME (153). They have found that *ex vivo* culturing and activation of T cells in a K^+^-elevated condition, which resembles the restricted nutrient uptake state in the K^+^-accumulated TME, prepares the T cells for the mentioned condition through their metabolic reprogramming (153). This preparation of T cells keeps their stemness and improves their antitumor cytolytic properties (153). Taken together, the abovementioned metabolic reprogramming strategies either improve T cell and CAR T cell responses, activity, and effector function in the TME or they avert the negative effects of particular TME-specific modifications performed by the tumor cells on the infiltrating T cells.

## Conclusion

30 years after the first genetic manipulation of T cells for generating CAR T cells, today, they can be known as the lifeblood of immunotherapeutics. In 2018, the American Society of Clinical Oncology (ASCO) named CAR T cell therapy “Advance of the Year” which further highlights the key role of this fighting soldier in the cancer treatment revolution. However, CAR T cell therapy toxicities and limitations appear as stones thrown at its fragile success. Therefore, clinical and basic science research efforts are highly required for addressing these ongoing/unsolved caveats. The herein discussed strategies might pave the way for less toxic and more effectual CAR T cell therapies with more favorable clinical outcomes since such toxicities are success-limiting factors themselves. However, as discussed throughout this review, each of these strategies might have its advantages and disadvantages over another which will define their applicability depending on the need. Moreover, the majority of these strategies are still in the laboratory or preclinical development which highlights the fact that they might require further optimization for translational purposes. Also, some of these strategies are currently under clinical evaluation and their clinical potential and efficacy are to be determined in the upcoming years. Furthermore, the fact that CAR T cell therapy has remarkably achieved four FDA approvals in the case of hematologic malignancies indicates that this type of immunotherapy might soon be a popular choice for the treatment of a wide spectrum of oncological indications (and even immunological indications). This should encourage scientists to optimize the already introduced strategies or to design and develop novel ones to address the remaining hindrances. In a nutshell, these strategies might be applied in a synergistic fashion to orchestrate a safer CAR T cell therapy whilst maximizing its tumoricidal efficacy in a way that it is just good news for patients with difficult-to-treat malignancies.

## Author Contributions

PooS: conceptualization, investigation, writing—original draft, writing—review and editing, validation, supervision. PouS: conceptualization, investigation, writing—original draft, writing—review and editing, validation, supervision. FR: writing—review and editing, validation, supervision. SK: writing—review and editing, validation. All authors contributed to the article and approved the submitted version.

## Conflict of Interest

The authors declare that the research was conducted in the absence of any commercial or financial relationships that could be construed as a potential conflict of interest.
